# CNN-Based Estimation of Sagittal Plane Walking and Running Biomechanics From Measured and Simulated Inertial Sensor Data

**DOI:** 10.3389/fbioe.2020.00604

**Published:** 2020-06-26

**Authors:** Eva Dorschky, Marlies Nitschke, Christine F. Martindale, Antonie J. van den Bogert, Anne D. Koelewijn, Bjoern M. Eskofier

**Affiliations:** ^1^Machine Learning and Data Analytics Lab, Department of Computer Science, Friedrich-Alexander University Erlangen-Nürnberg (FAU), Erlangen, Germany; ^2^Mechanical Engineering Department, Cleveland State University, Cleveland, OH, United States

**Keywords:** biomechanics, biomechanical simulation and analysis, gait analysis, musculoskeletal simulation, inertial sensors, optimal control, machine learning, convolutional neural networks - CNN

## Abstract

Machine learning is a promising approach to evaluate human movement based on wearable sensor data. A representative dataset for training data-driven models is crucial to ensure that the model generalizes well to unseen data. However, the acquisition of sufficient data is time-consuming and often infeasible. We present a method to create realistic inertial sensor data with corresponding biomechanical variables by 2D walking and running simulations. We augmented a measured inertial sensor dataset with simulated data for the training of convolutional neural networks to estimate sagittal plane joint angles, joint moments, and ground reaction forces (GRFs) of walking and running. When adding simulated data, the root mean square error (RMSE) of the test set of hip, knee, and ankle joint angles decreased up to 17%, 27% and 23%, the RMSE of knee and ankle joint moments up to 6% and the RMSE of anterior-posterior and vertical GRF up to 2 and 6%. Simulation-aided estimation of joint moments and GRFs was limited by inaccuracies of the biomechanical model. Improving the physics-based model and domain adaptation learning may further increase the benefit of simulated data. Future work can exploit biomechanical simulations to connect different data sources in order to create representative datasets of human movement. In conclusion, machine learning can benefit from available domain knowledge on biomechanical simulations to supplement cumbersome data collections.

## 1. Introduction

Due to technological advances in wearable computing, it is now possible to measure human movement outside the lab, in the natural environment (Seshadri et al., 2019). This facilitates a continuous monitoring of patients and athletes supporting medical diagnosis, performance assessment in sports, prevention of falling or sport-related injuries, tracking of disease progression and evaluating the efficiency of treatment. Extracting useful information from sensor data remains challenging as uncontrolled natural conditions imply variations in sensor placement, in data quality, and a wide range of movement patterns. Typically, only discrete variables are computed from sensor data, such as speed, stride length, and step frequency (Hannink et al., 2017; Falbriard et al., 2018; Zrenner et al., 2018). However, a comprehensive biomechanical analysis, which involves the evaluation of joint angles, joint moments, muscle forces, and ground reaction forces (GRFs), would be beneficial to gain a deeper understanding of the movement mechanics and underlying causes.

However, low-quality sensor data and sparse measurements make it difficult to achieve a comprehensive analysis that is comparable to laboratory results, where optical motion capture (OMC) systems and force plates are available. Different methods were developed to address the challenge of extracting the kinematic and kinetic parameters of movements from sensor data, commonly inertial sensor data. These methods can be divided into physics-based or data-driven approaches.

Physics-based approaches use kinematic chain models or musculoskeletal models in combination with Kalman filters or global optimization to constrain the solution space (Roetenberg et al., 2009; Koning et al., 2013; Kok et al., 2014; Miezal et al., 2017; Karatsidis et al., 2018; Dorschky et al., 2019). Physical models can act as a filter to the noisy sensor data. Moreover, reconstructing the movement with a musculoskeletal model yields a comprehensive analysis including muscle forces, kinematics, and kinetics. In contrast to data-driven approaches, no lab measurements are necessary to train the model. However, global optimization methods require a relatively high computation time (Kok et al., 2014; Dorschky et al., 2019) and are thus less suitable for real-time applications. In addition, model inaccuracies such as simplified ground contact lead to errors in GRF and joint moment estimations.

Data-driven approaches can directly learn a mapping between sensor data and target biomechanical variables based on lab measurements (Wouda et al., 2018; Komaris et al., 2019; Stetter et al., 2019; Zell and Rosenhahn, 2019). Machine learning algorithms can reveal hidden relationships between sensor data and biomechanical variables, in particular, deep learning is a promising approach to model time series data of human movement (Halilaj et al., 2018). Trained models can be exploited in real-time to provide instantaneous feedback to the patient, athlete, or coach. For example, an early warning system monitoring the internal joint loads during sports could potentially prevent catastrophic non-contact knee injuries (Johnson et al., 2019). Furthermore, low-latency feedback on joint moments could help gait retraining in osteoarthritis patients to reduce the knee adduction moment (Preece et al., 2009). However, training data-based models requires a representative dataset, which is cumbersome to acquire as it typically involves synchronized recordings of inertial sensors and OMC systems. It is often impractical to collect a dataset large enough to train deep neural networks. Variations in movement patterns, different sensor positions, and movement or sensor artifacts can lead to high generalization errors within data-based models (Wouda et al., 2018).

Strategies like data augmentation and transfer learning have been applied to improve robustness and generalization of data-based models. Um et al. (2017) used label-preserving transformations of the sensor data (e.g., rotations, permutations, and time-warping) to augment the training dataset. This improved the robustness of the model with respect to sensor position and noise, but did not account for variations in movement patterns as the target variables remained unchanged. Veiga et al. (2017) and Johnson et al. (2019) utilized pre-trained deep neural networks from the image domain as a feature extractor. The former authors used images showing line curves of sensor signals. However, characteristic features of one dimensional inertial sensor signals likely differ from photographic images extracted from the ImageNet database. Johnson et al. (2019) transformed the data of five accelerometers into two-dimensional images: one dimension representing the sensor locations and the other dimension the normalized time. The acceleration magnitude was quantized to greyscale or RGB colorspace, what probably caused information loss.

To learn from sufficient data and incorporate variations of movement, Johnson et al. (2019) synthesized accelerometer data via double-differentiation of marker trajectories from their OMC archive. Huang et al. (2018) also synthesized inertial sensor data from motion capture datasets using a 3D model of the human body shape and pose (SMPL) together with a virtual sensor model. Mundt et al. (2020a,b) used OMC data from several studies of their lab together with a biomechanical model to create a large simulated dataset, which was used for training feedforward neural networks to estimate joint kinematics and kinetics. One drawback of these approaches is that additional datasets containing OMC data or SMPL poses of the movement of interest were required. Notably, Huang et al. (2018) reported that combining these datasets was non-trivial. Moreover, each recorded motion trajectory led to only one synthetic sensor trajectory. An infinite number of random samples can be generated using statistical modeling. Norgaard et al. (2018) synthesized inertial sensor data from random vectors using a generative adversarial network. Their approach did not include biomechanical constraints to extract physically plausible samples.

Our goal is to use physical knowledge of biomechanics to alleviate the issue of data limitation. We contribute a new method to expand a training dataset via biomechanical simulations created by solving optimal control problems. We simulated musculoskeletal models to follow walking and running trajectories which were randomly sampled from a “small” measured training dataset. In principle, an infinite number of simulations could be obtained with matching inertial sensor data and biomechanical variables. The constraints in the optimal control problem ensured that simulated motions were physically possible and dynamically consistent.

We evaluated if learning on simulated data can decrease generalization errors, how much simulated data is necessary, and what happens in the case of even smaller training datasets. Therefore, we trained convolutional neural networks (CNNs) to map inertial sensor data of walking and running cycles to joint angles, joint moments and GRFs. We compared the performance of the CNNs for training on only measured data with training on measured and simulated data.

## 2. Materials and Methods

**Figure 1:**
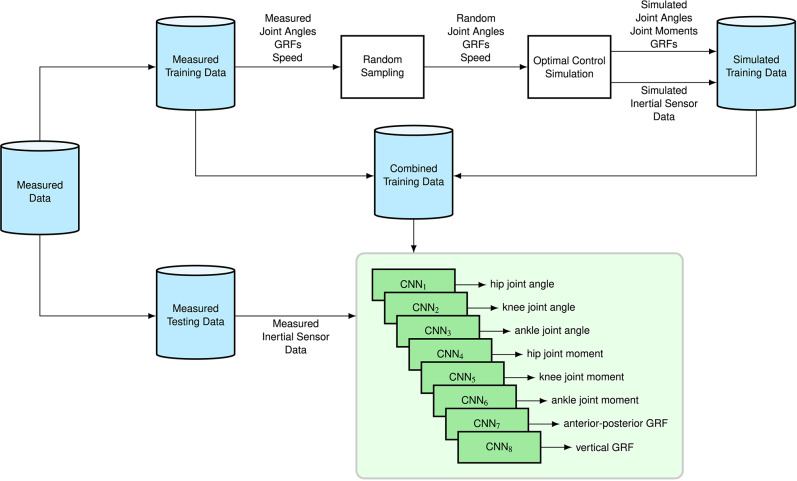
We trained CNNs to estimate sagittal lower body kinematics and kinetics from accelerometer and gyroscope data from four inertial sensors which were placed on the lower body. Therefore, we created simulated data based on the measured training dataset (described in section 2.1): we drew random samples from measured joint angles, GRFs, and walking/running speeds (see section 2.2), which were then tracked by musculoskeletal models solving optimal control problems (see section 2.3). Simulated movements yielded biomechanics with matching inertial sensor data using a virtual inertial sensor model.

Figure 1 shows the overview of the proposed methods. We trained CNNs (LeCun et al., 1989) to estimate sagittal lower body kinematics and kinetics from accelerometer and gyroscope data from four inertial sensors which were placed on the lower body. Therefore, we created simulated data based on the measured training dataset (described in section 2.1): we drew random samples from measured joint angles, GRFs, and walking/running speeds (see section 2.2), which were then tracked by musculoskeletal models solving optimal control problems (see section 2.3). Simulated movements yielded biomechanics with matching inertial sensor data using a virtual inertial sensor model. We explain the network architecture of the CNNs in section 2.4 and the evaluation process in section 2.5.

### 2.1. Measured Data

**Figure 2:**
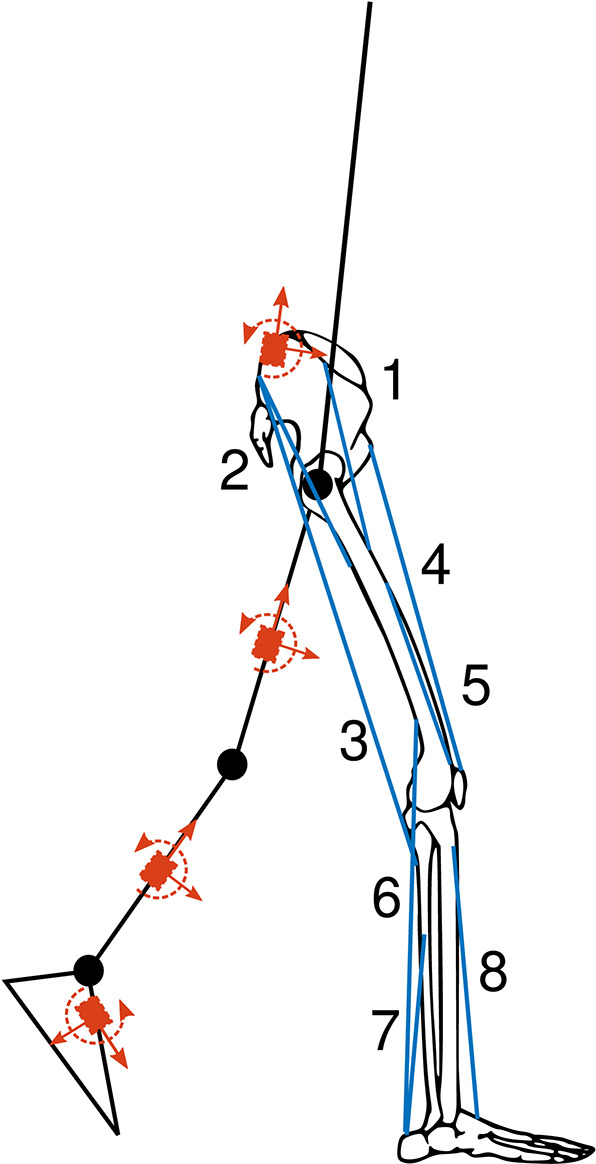
Conceptual drawing of musculoskeletal model consisting of seven rigid segments and 16 Hill-type muscles (blue) with seven virtual inertial sensors (red). The muscles are drawn for the right leg only: 1—iliopsoas, 2—glutei, 3—hamstrings, 4—rectus femoris, 5—vasti, 6—gastrocnemius, 7—soleus, and 8—tibialis anterior. The virtual sensors are drawn for the left leg only simulating sagittal inertial sensor signals: anterior-posterior accelerations, longitudinal accelerations, and medial-lateral angular velocities indicated with red arrows. The figure is taken and modified from Dorschky et al. (2019).

We used the data recorded by Dorschky et al. (2019), which consisted of data from 10 subjects (denoted by S01-S10) walking and running at six different speeds with 10 trials each. The walking speeds were: 0.9 to 1.0 m s^-1^, 1.2 to 1.4 m s^-1^, and 1.8 to 2.0 m s^-1^. The running speeds were: 3.1 to 3.3 m s^-1^, 3.9 to 4.1 m s^-1^, and 4.7 to 4.9 m s^-1^. The dataset comprises 595 (valid) walking and running cycles in total. It includes data from seven custom-built inertial sensors (Portabiles GmbH, Erlangen, DE) (Blank et al., 2015) including tri-axial accelerometers (±16 g) and gyroscopes (±2.000 deg/s) sampled at 1.000 Hz. Corresponding lower body joint angles, moments, and GRFs in the sagittal plane were computed from data measured with an OMC system with 16 infrared cameras (Vicon MX, Oxford, UK) and one force plate (Kistler Instruments Corp, Winterhur, CH), which were sampled at 200 and 1,000, respectively. The speed was measured by two light barriers at a distance of 2 m. In order to analyze right-sided biomechanics, data from four inertial sensors were used; located at the lower back, the lateral right thigh, the lateral right shank, and over the 2nd to 4th metatarsal of the right foot. Sensor positions are shown in Figure 2. Sensor data was aligned with segmental axes based on calibrating movements. Eight sagittal plane biomechanical variables were used as a reference: the right-side hip, knee, and ankle flexion angles and moments, and the anterior-posterior (A-P) and vertical GRFs. Biomechanical variables and sensor data were segmented into isolated segments of data from initial contact to initial contact and resampled to 100 time points using linear interpolation. For evaluation in section 2.5, the data from three subjects (S01, S02, and S03) were left out for testing and the data of the remaining subjects (S04-S10) were used for training the CNNs. Simulated data was created from the measured biomechanics of the training subjects.

### 2.2. Random Sampling

We estimated the joint distribution of measured joint angles, GRFs, and walking and running speeds of individual training subjects and drew random samples from these distributions. To achieve this, we concatenated for each walking and running cycle the 100 time points of right-sided hip, knee, and ankle joint angle and the A-P and vertical GRF and the corresponding speed. Thus, every walking and running cycle was described by a vector of ℝ^501^. For each subject *S*_*i*_, the vectors of the (approximately) 30 walking and 30 running cycles were stacked to matrices of ℝ^30×501^, **Z**_*S*_*i*_,walking_ and **Z**_*S*_*i*_,running_, whose rows represented observations of the random variable vectors **z**_*S*_*i*_,walking_ and **z**_*S*_*i*_,running_, respectively. We assumed multivariate normal distributions: ${\text{z}}_{S_{i},\text{walking}}∼N\left(\mu_{S_{i},\text{walking}},\Sigma_{S_{i},\text{walking}}\right)$ and ${\text{z}}_{S_{i},\text{running}}∼N\left(\mu_{S_{i},\text{running}},{\text{Σ}}_{S_{i},\text{walking}}\right)$. Therefore, we computed the sample means **μ**_*S*_*i*_,walking_ and $\mu_{S_{i},\text{running}}\in {\mathbb{R}}^{501}$ over the rows of **Z**_*S*_*i*_,walking_ and **Z**_*S*_*i*_,running_ and the sample covariance matrices **Σ**_*S*_*i*_,walking_ and $\Sigma_{S_{i},\text{running}}\in {\mathbb{R}}^{501\times 501}$ estimating the covariance between the random variables (the columns of **Z**_*S*_*i*_,walking_/**Z**_*S*_*i*_,running_). We drew 1,000 random samples from each distribution to serve as tracking data for the optimal control simulation in section 2.3 using Matlab R2018a mvnrnd function (Kotz et al., 2004). Random samples of **z** were partitioned into joint angles, GRFs, and speed. Joint angles and GRFs were parted in the middle such that they could be used as tracking data for the right and left leg, as only a half symmetric cycle was simulated.

### 2.3. Simulated Data

We created seven planar musculoskeletal models (Van den Bogert et al., 2012), one for each of the training subjects. Each musculoskeletal model consisted of seven rigid segments (trunk, thighs, shanks, and feet) connected by six hinge joints (hip, knee, ankle in each limb) resulting in nine kinematic degrees of freedom. In addition, each model had 16 Hill-type muscles which are shown in Figure 2. The segments of the model were scaled using the bodyweight (BW) and bodyheight (BH) of each subject according to Winter (2009). The multi-body dynamics and muscle dynamics are described in previous publications (Van den Bogert et al., 2011; Dorschky et al., 2019). The unknowns of the model, which were the generalized coordinates and velocities, the muscle activations, muscle lengths, and the contact state, were summarized in state vector **x**(*t*). The control vector **u**(*t*) described the neural excitations of the muscles at time *t*. The model was simulated to follow random trajectories *m*(*t*) of the right and left hip, knee, and ankle angles and anterior-posterior and vertical GRFs while minimizing average muscular effort. We simulated a half walking/running cycle of duration *T* assuming left-right symmetry, to speed up simulation. The simulation was formulated as the following optimal control problem:

(1a) $$\begin{array}{l} \underset{\text{x}(t),\text{u}(t)}{\text{minimize}}\;J(\text{x}(t),\text{u}(t))\\ \text{​}=\frac{1}{T}\int_{0}^{T}\left(\underset{\text{track random trajectories}}{\underset{︸}{\frac{1}{10}\sum_{j=1}^{10}\frac{{\left(s_{j}(t)-m_{j}(t)\right)}^{2}}{\sigma_{j}{\left(t\right)}^{2}}}}+\underset{\text{muscular effort}}{\underset{︸}{\frac{W_{\text{effort}}}{16}\sum_{i=1}^{16}u_{i}{\left(t\right)}^{2}}}\right)\text{d}t\\ \text{​​}+W_{\text{reg}}J_{\text{reg}} \end{array}$$

(1b) $$\begin{array}{l} \text{subject to}\\ {\text{x}}_{L}\leq \text{x}\leq {\text{x}}_{U} \end{array}$$

(1c) $${\text{u}}_{L}\leq \text{u}\leq {\text{u}}_{U}$$

(1d) $$\text{f}(\text{x}(t),\dot{\text{x}}(t),\text{u}(t))=0$$

(1e) $$\text{x}(0)+vT{\text{e}}_{x}-{\text{x}}^{*}(T)=0.$$

The objective function J(x(t),u(t)) consisted of a tracking, an effort, and a regularization term with the weights *W*_effort_ = 0.1 and *W*_reg_ = 0.00001. The weighting was chosen empirically so that tracking and effort term had about the same magnitude and the regularization term was of lower magnitude. In the tracking term, the quadratic deviation of simulated trajectory *s*(*t*) to the prescribed trajectory *m*(*t*), normalized to the measured variance σ(*t*), was minimized. Average muscular effort, the mean squared value of muscle excitations, was minimized to resolve muscle ambiguity and to allow the model to deviate from the random trajectories finding a more efficient and potentially more natural movement path. In the regularization term, $J_{\text{reg}}$, the integral of the sum of squares of the time derivatives of all state and control variables was minimized helping the optimization to converge more quickly.

Equations (1b) and (1c) were the lower (L) and upper (U) bounds of the state vector **x** and the control vector **u** ∈ [0, 5] [the same bounds as in Dorschky et al. (2019)]. Dynamic equilibrium was constrained in Equation 1d. To do so, the dynamic equations, which were the multi-body dynamics, muscle dynamics, and contact dynamics (Van den Bogert et al., 2011; Dorschky et al., 2019), were formulated implicitly. In constraint Equation 1e, we enforced symmetry of the right and left leg with a forward translation in direction **e**_**x**_, where *v* is the randomly sampled speed (see section 2.2) and x^*^ is the mirrored state vector of the right and left leg. The optimal control problem, Equation (1), was solved using direct collocation. The state and control vector were sampled to 50 time points using the Backward Euler method. We used the open source optimizer IPOPT (Wächter and Biegler, 2006) and ran the simulations on a high performance cluster.

The simulation results were expanded to a whole symmetric walking/running cycle with 100 time points. We used the simulated biomechanics of the right leg for training the CNNs in section 2.5. Given the simulated movements, we could extract accelerometer and gyroscope signals at any position of the models. In this work, we used the measured sensor position for each subject from section 2.1 and calculated virtual inertial sensor data as introduced in Dorschky et al. (2019). Gyroscope signals were computed from global trunk orientation and relative joint angular rates. Accelerometer signals were computed from the segment accelerations adding gravity and centrifugal acceleration dependent on sensor position.

### 2.4. Convolutional Neural Network

We trained CNNs to learn a mapping between inertial sensor data and sagittal plane biomechanical variables for walking/running cycle defined from initial contact to initial contact sampled at 100 time points. The sampling was chosen to match the simulated data. We trained eight separate CNNs, one for each output variable, namely the right hip, knee, and ankle angles and moments and A-P and vertical GRFs. As input, we used the sagittal plane sensor data of the hip sensor, right thigh sensor, right shank sensor and right foot sensor. We used two accelerometer axes (A-P and longitudinal) and one gyroscope axis (medial-lateral) of each sensor, resulting in an input dimension of 100 × 12. We scaled the data using min-max normalization.

The CNN architecture is based on previous work performing gait analysis from inertial sensor data of segmented strides (Hannink et al., 2017; Zrenner et al., 2018). They used two or three 1D convolutional layers to extract temporal features from accelerometer and gyroscope data. We found that 2D convolutional layers filtering over time and sensor channels were superior to 1D convolutional layers performing just temporal convolutions. They estimated single spatio-temporal gait parameters instead of biomechanical variables over gait cycles. Thus, the number of output nodes was adapted to 100 time points in our work.

Table 1 provides an overview of the network, which consisted of two convolutional layers for feature extraction with zero padding, a stride length of one, and a rectified linear activation function. After each convolutional layer, max-pooling was applied. Two convolutional layers seemed to yield superior performance in comparison to one or three convolutional layers because underfitting occurred in the first case and overfitting in the other case. The data was flattened before passing it to two dense layers for non-linear multivariate regression. The first dense layer had a non-linear rectified linear activation function and 100 nodes. The output layer was a dense layer with linear activation function and 100 nodes. To prevent the model from overfitting, we used L2 kernel regularization. During cross-validation (CV), we inspected the learning curves for overfitting verifying that the validation error did not increase with the number of iterations. We used the ADAM optimizer (Kingma and Ba, 2015) and the mean squared error loss function to train the CNNs. The batch size, learning rate, number of epochs, and L2 regularization factor were empirically set based on the measured training dataset considering specifically the values in Table 2. The number of filters, kernel size, and max-pooling were tuned using leave-one-subject-out CV within the seven training subjects (S4-S10) testing the hyperparameters in Table 1. The network was implemented in Python using Keras with Tensorflow backend (Chollet, 2015; Abadi et al., 2016). Our implementation of the CNN can be found in the Supplementary Material.

**Table 1:** Architecture of convolutional neural networks with tuned hyperparameters.

**Layer**	**Name**	**Hyperparameter**	**Search space**	**Selected value**	**Size of output**
1	Convolution-ReLU	Kernel_size1, filters1	{3×1, 5×1, 7×1, 3×3, 5×3, 7×3}×{8, 16, 32, 64, 128}	5×3, 64	100×12×64
2	Max-Pooling	Pool_size1	{2×1, 2×2}	2×2	50×6×64
3	Convolution-ReLU	Kernel_size2, filters2	{3×1, 5×1, 7×1, 3×3, 5×3, 7×3}×{16, 32, 64, 128, 256}	5×3, 128	50×6×128
4	Max-Pooling	Pool_size2	{2×1, 2×2}	2×2	25×3×128
5	Flattening	-	-	-	9600
6	Dense-ReLU	-	-	-	100
7	Dense	l2_reg	{0.01,0.001,0.0001}	0.001	100

**Table 2:** Hyperparameters related to training the convolutional neural networks.

**Parameter**	**Considered values**	**Selected value**
Batch size	{32,64,128,256,516}	64
Learning rate	{0.01,0.001,0.0001}	0.001
Number of epochs	{500,1000,2000,3000}	1000

### 2.5. Evaluation

The chosen hyperparameters were fixed for all further evaluations. We trained every CNN with 10 random seeds to test the robustness of results with respect to different random samples of simulated data and random initializations of CNN layers. For comparison purposes, we used the same random seeds for all different training sets. First, we trained the CNNs using only measured data of subjects S04-S10 (training dataset) and tested them with the data of subjects S01-S03 (test dataset). Then, we evaluated how simulated data influences the resulting evaluation metrics. Therefore, we subsequently added simulated data to the training dataset (418 samples) to obtain twice (836 samples), four times (1,672 samples), eight times (3,344 samples), and 16 times (6,688 samples) the amount of training samples. Simulated data was picked randomly and equally from the 1,000 simulations of each training subject of the walking and running simulations. Thus, the same amount of simulated data was taken from each normal distribution in section 2.2. We used the Python's random module to randomly pick simulated data (Matsumoto and Nishimura, 1998). As we trained every CNN 10 times with different random samples, we made sure that results were robust to random sampling. We trained the networks jointly on simulated and measured training data, which was randomly shuffled at each epoch.

Secondly, we evaluated the model when using less training subjects. We used only four subjects (S07-S10) and two subjects (S09 and S10) for training and tested it with the same three test subjects (S01-S03). For each amount of training subjects, we expanded the respective measured dataset to obtain twice, four times, eight times, and 16 times the amount of training samples. The simulated data was used from the training subjects only: from four subjects (S07-S10) and two subjects (S09 and S10), respectively.

For evaluation, we computed the root mean square error (RMSE) and the Pearson correlation coefficient between estimated biomechanics and reference biomechanics. The RMSE was expressed in degrees for joint angles, in BW times BH in percent for joint moments, and in BW percent for GRFs. GRFs were only evaluated over the stance phase using the time points from force plate measurements. For evaluating individual results, performance metrics were computed using all 100 samples of all walking and running cycles of each individual subject and the results were averaged over the 10 random seeds. We used the Fisher-transform to estimate the mean of the Pearson correlation coefficient. For evaluating overall results, performance metrics were computed using all test samples without separating the results of individual subjects and averaged over the 10 random seeds.

## 3. Results

**Figure 3:**
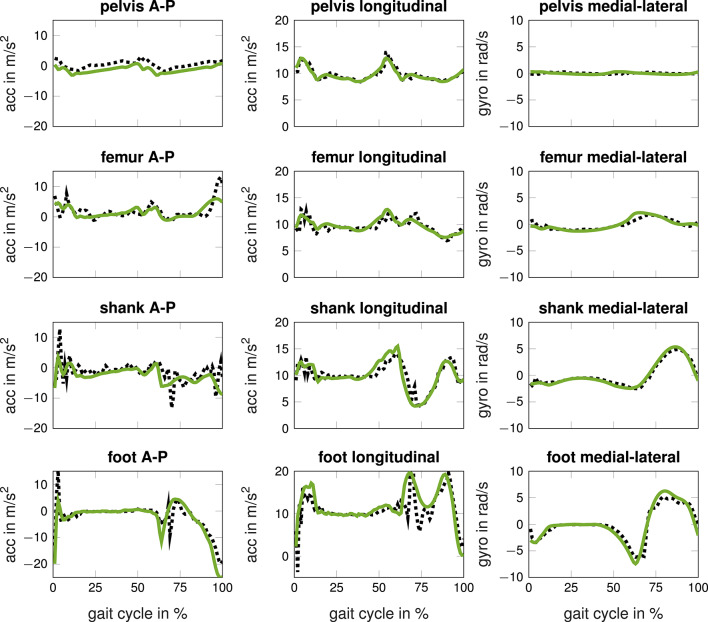
Measured (black dotted) and simulated (green solid) accelerometer (acc) and gyroscope (gyro) data in the sagittal-plane of one subject running at fast speed. The inertial sensors were located at the lower back, the lateral right thigh, the lateral right shank, and at the span of the right foot.

**Figure 4:**
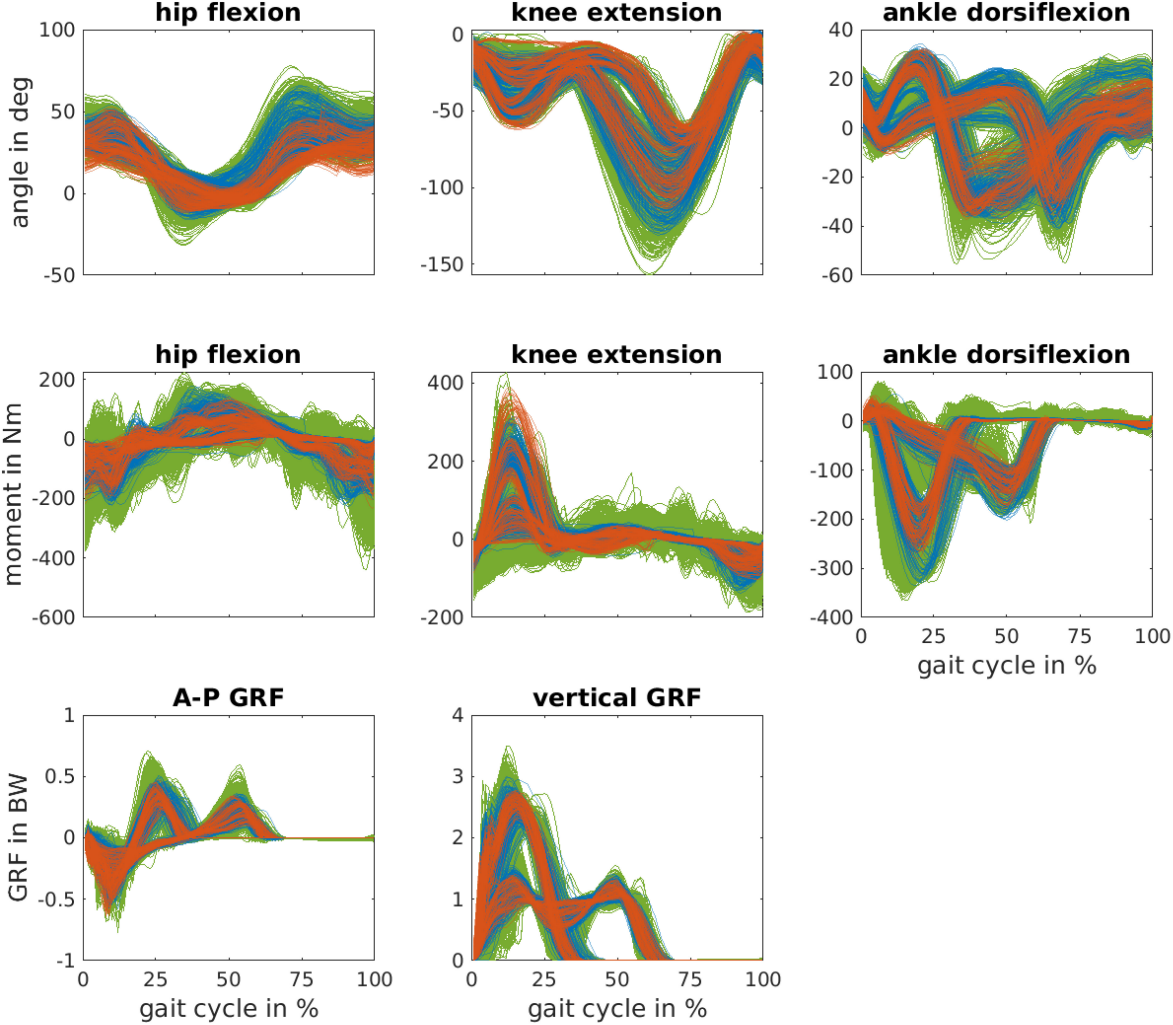
Simulated biomechanics data (green) created from a measured training dataset of seven subjects (blue). Simulated and measured data were used to train data-based models which were tested using the measured data of three independent subjects (red). The anterior-posterior (A-P) and vertical ground reaction force (GRF) are normalized to the bodyweight (BW) of each subject.

Each simulation had a mean CPU time of (3.6 ± 2.0) on Intel Xeon processors E3-1240, whereas multiple simulations ran in parallel on a cluster. Figure 3 shows the simulated inertial sensor data and the corresponding measured data. The pattern is similar, while the simulated data is smoother than the measured data. Figure 4 shows the joint angles, moments, and GRFs of measurements and simulations used for training and the measured data used for testing. The simulated data covers a wider range than measured data and is more dense. The simulated joint moments show more oscillations, especially in the hip flexion moment. Testing data lies outside of the training data distribution for hip joint angle for S01, knee angle during stance for S02 and peak knee moment for S02.

Training all CNNs including the hyperparameter search took about two weeks on a Nvidia GeForce GTX 1080 Ti. However, inference time of each CNN was less than 1ms per gait cycle.

Tables 3, 4 summarize the individual results of the test subjects for training with the data of all seven training subjects and a different amount of simulated data. In addition, the results of the leave-one-subject-out CV of the seven training subjects are presented using the selected hyperparameters from Tables 1, 2. For all three test subjects, the performance of the CNNs for joint angles increased adding simulated data to the training dataset. The estimation of the hip joint moment was best without using simulated data. Simulated data improved the RMSE of the knee joint moment for all test subjects, whereas the Pearson correlation coefficient only slightly improved for test subjects S01 and S03. The A-P and vertical GRF improved for test subject S01 and S02 adding simulated data, while the performance decreased for test subject S03. Adding more simulated data led to a decrease in performance. Looking at results of the CV, the RMSE of joint angles is lower and Pearson correlation coefficients are higher when simulated data is added. Simulated data did not increase performance for joint moments and vertical GRFs in the CV.

**Table 3:** The root mean square error (RMSE) of sagittal plane joint angles, joint moments, and anterior-posterior (A-P) and vertical ground reaction force (GRF) is presented for varying ratios between simulated (sim) and measured (meas) data.

	**Sim/meas**	**Hip angle**	**Knee angle**	**Ankle angle**	**Hip moment**	**Knee moment**	**Ankle moment**	**A-P GRF**	**Vertical GRF**
	**data**	**degree**	**degree**	**degree**	**BWBH%**	**BWBH%**	**BWBH%**	**BW%**	**BW%**
CV	0	5.38 (1.57)	5.22 (1.22)	5.50 (1.64)	**1.62 (0.23)**	**1.14 (0.13)**	**1.32 (0.42)**	**4.33 (0.48)**	**14.44 (2.08)**
	1	5.19 (1.36)	4.95 (1.38)	5.00 (1.52)	1.66 (0.20)	1.21 (0.08)	1.38 (0.42)	4.36 (0.49)	14.75 (4.58)
	3	**5.08 (1.77)**	**4.81 (1.19)**	4.86 (1.53)	1.75 (0.23)	1.27 (0.08)	1.35 (0.39)	4.51 (0.31)	15.28 (3.55)
	7	5.17 (1.44)	5.09 (1.65)	4.72 (1.35)	1.76 (0.29)	1.35 (0.16)	1.36 (0.37)	4.35 (0.41)	15.10 (3.06)
	15	5.37 (1.57)	4.93 (1.20)	**4.60 (1.32)**	1.78 (0.30)	1.28 (0.15)	1.39 (0.34)	4.63 (0.52)	16.07 (3.75)
S01	0	9.42 (0.48)	4.45 (0.41)	3.29 (0.25)	**1.71 (0.11)**	1.21 (0.12)	**0.88 (0.12)**	4.52 (0.26)	11.74 (0.88)
	1	8.98 (0.59)	4.28 (0.55)	3.54 (0.47)	1.88 (0.12)	**1.07 (0.07)**	0.98 (0.07)	4.70 (0.28)	10.46 (0.86)
	3	9.11 (0.26)	3.87 (0.31)	3.23 (0.38)	1.97 (0.13)	1.31 (0.12)	1.00 (0.12)	4.23 (0.18)	**9.99 (0.76)**
	7	8.94 (0.55)	3.57 (0.27)	3.49 (0.23)	2.01 (0.10)	1.30 (0.11)	1.03 (0.10)	4.22 (0.13)	12.33 (0.80)
	15	**8.77 (0.49)**	**3.31 (0.34)**	**2.87 (0.30)**	2.07 (0.11)	1.36 (0.15)	1.05 (0.10)	**3.76 (0.31)**	13.53 (1.04)
S02	0	6.49 (0.59)	10.44 (1.31)	4.40 (0.57)	**1.44 (0.10)**	2.06 (0.25)	1.86 (0.21)	4.41 (0.38)	13.24 (1.20)
	1	6.32 (0.89)	8.69 (0.49)	4.24 (0.28)	1.71 (0.20)	**2.04 (0.21)**	**1.59 (0.14)**	**4.03 (0.46)**	**12.16 (0.77)**
	3	5.39 (0.57)	7.70 (0.44)	4.24 (0.46)	1.81 (0.06)	2.08 (0.20)	1.67 (0.15)	4.21 (0.18)	13.67 (1.01)
	7	4.47 (0.46)	**7.26 (0.45)**	4.47 (0.32)	1.89 (0.10)	2.27 (0.13)	1.78 (0.15)	4.40 (0.30)	12.42 (0.83)
	15	**3.69 (0.19)**	7.29 (0.40)	**4.18 (0.50)**	1.95 (0.12)	2.39 (0.25)	1.73 (0.17)	4.21 (0.15)	15.34 (1.21)
S03	0	3.71 (0.24)	5.52 (0.56)	6.31 (0.49)	**1.32 (0.04)**	1.96 (0.08)	**1.05 (0.07)**	**4.29 (0.23)**	**12.91 (0.62)**
	1	3.43 (0.31)	4.82 (0.39)	4.43 (0.24)	1.61 (0.13)	1.76 (0.15)	1.11 (0.03)	5.10 (0.29)	13.75 (0.93)
	3	3.10 (0.18)	4.47 (0.23)	4.30 (0.31)	1.62 (0.07)	1.79 (0.09)	1.24 (0.11)	4.36 (0.25)	14.25 (1.17)
	7	**3.00 (0.14)**	**4.36 (0.34)**	4.01 (0.35)	1.72 (0.09)	**1.75 (0.13)**	1.20 (0.09)	4.83 (0.27)	14.75 (0.72)
	15	3.06 (0.19)	4.62 (0.12)	**3.94 (0.38)**	1.78 (0.11)	1.77 (0.08)	1.21 (0.04)	4.95 (0.21)	16.24 (1.08)

*Joint moments and GRFs are normalized to bodyweight (BW) and bodyheight (BH). The first rows show the mean RMSE and its standard deviation of the leave-one-subject-out cross-validation (CV) on the training dataset for the chosen hyperparameter. The subsequent rows show the mean RMSE and standard deviation over 10 random seeds for the three test subjects S01-S03 using the data of seven subjects for training. Bold highlighting indicates the lowest mean value in the respective column*.

**Table 4:** The Pearson correlation coefficient of sagittal plane joint angles, joint moments, and anterior-posterior (A-P) and vertical ground reaction force (GRF) is presented for varying ratios between simulated (sim) and measured (meas) data.

	**Sim/meas**	**Hip angle**	**Knee angle**	**Ankle angle**	**Hip moment**	**Knee moment**	**Ankle moment**	**A-P GRF**	**Vertical GRF**
	**data**								
CV	0	0.969	0.989	0.962	**0.940**	**0.975**	**0.981**	0.970	**0.980**
	1	**0.974**	**0.990**	0.967	0.937	0.969	0.974	**0.971**	0.979
	3	0.973	**0.990**	0.970	0.931	0.964	0.974	0.968	0.977
	7	0.973	0.990	0.972	0.931	0.958	0.972	0.970	0.977
	15	0.973	**0.990**	**0.975**	0.927	0.958	0.971	0.967	0.975
S01	0	0.953	0.991	0.975	**0.920**	0.976	**0.985**	0.979	0.988
	1	0.958	0.990	0.981	0.899	**0.977**	0.983	0.980	**0.991**
	3	0.960	0.992	0.985	0.900	0.970	0.983	**0.982**	**0.991**
	7	**0.962**	0.993	0.985	0.880	0.968	0.982	0.980	0.989
	15	0.959	**0.994**	**0.987**	0.865	0.965	0.982	0.980	0.985
S02	0	0.970	0.989	0.962	**0.948**	**0.947**	**0.982**	0.972	0.979
	1	0.972	0.990	0.969	0.932	0.946	0.979	0.966	**0.983**
	3	**0.975**	0.990	0.975	0.938	0.941	0.973	0.971	0.980
	7	**0.975**	0.992	0.978	0.935	0.946	0.972	0.973	0.981
	15	**0.975**	**0.993**	**0.980**	0.936	0.941	0.972	**0.974**	0.980
S03	0	0.975	0.982	0.910	**0.948**	0.979	**0.978**	**0.976**	**0.981**
	1	0.982	0.988	0.941	0.924	**0.981**	0.970	0.970	**0.981**
	3	**0.984**	0.990	0.940	0.918	0.977	0.962	0.971	0.976
	7	0.983	**0.991**	0.948	0.906	0.974	0.961	0.969	0.974
	15	0.982	0.990	**0.949**	0.899	0.973	0.959	0.966	0.974

*The first rows show the mean Pearson correlation coefficient of the leave-one-subject-out cross-validation (CV) on the training dataset for the chosen hyperparameter. The subsequent rows show the mean Pearson correlation coefficient over ten random seeds for the three test subjects S01-S03 using the data of seven subjects for training. Bold highlighting indicates the lowest mean value in the respective column*.

**Figure 5:**
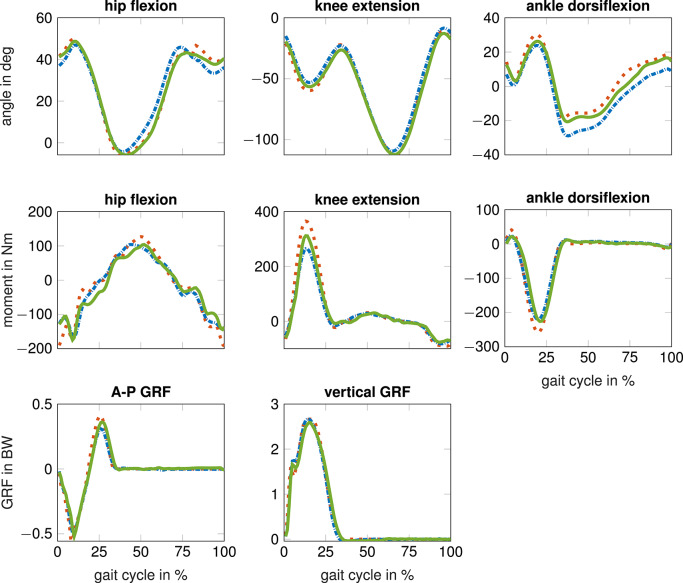
Results for test subject S03 running at fast speed: reference biomechanics from optical motion capturing (dotted red) compared to estimated biomechanics from inertial sensor data using no simulated data (blue dashed dotted) and seven times as much simulated as measured data (green solid). The anterior-posterior (A-P) and vertical ground reaction force (GRF) are normalized to the bodyweight (BW).

Figure 5 shows the estimated biomechanics for S03 running at fast speed using no simulated data and using seven times more simulated than measured data. The estimated hip angle, ankle angle, and knee moment are closer to the reference when simulated data was added to the training dataset. For example, the peak knee extension moment is higher and the estimated ankle angle is closer to the reference during swing phase.

**Figure 6:**
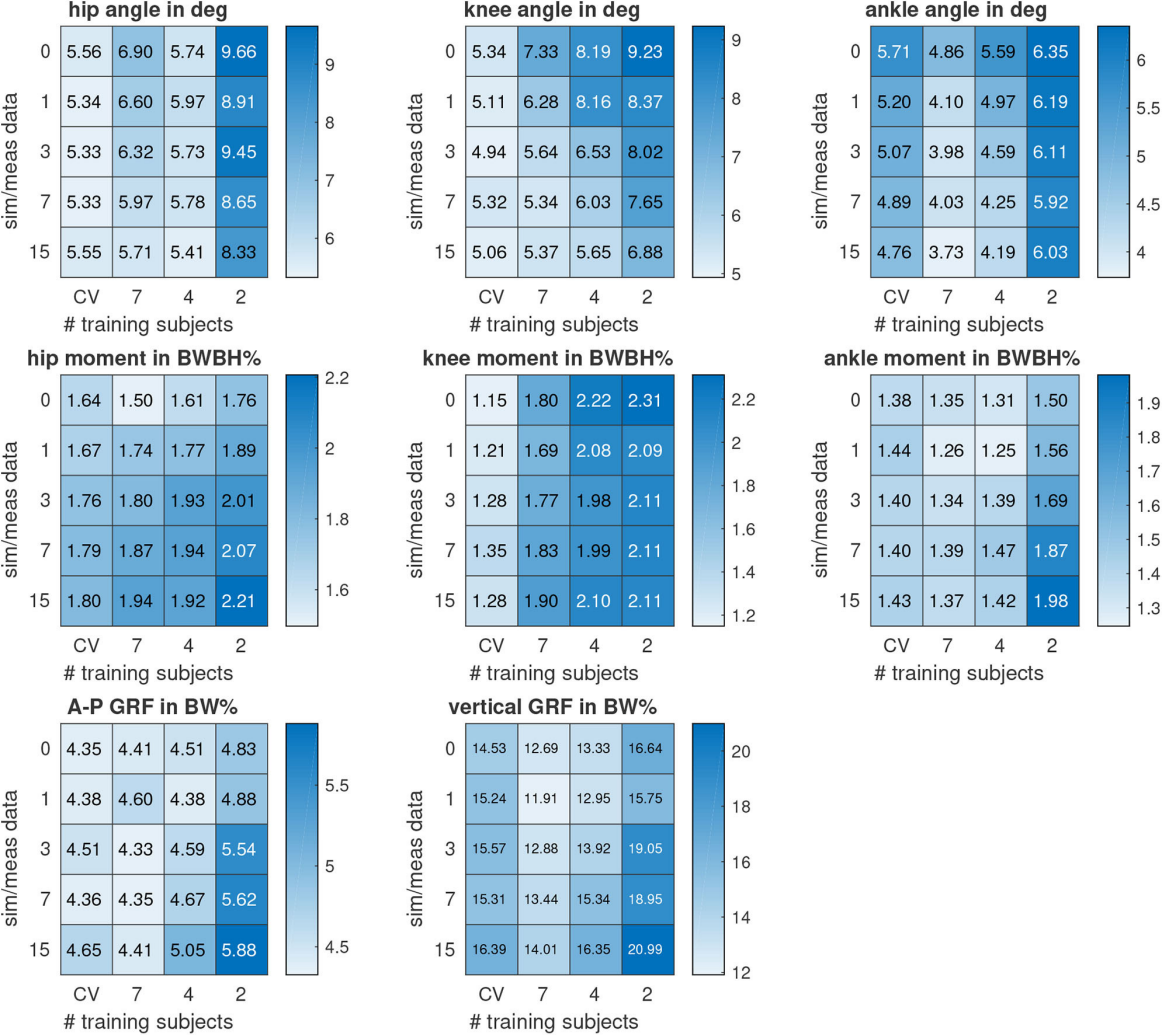
Overall results of the root mean square error (RMSE) for the estimated sagittal plane biomechanical variables. The vertical axis indicates the ratio between simulated (sim) and measured (meas) data used for training. The horizontal axis indicates the number of training subjects whose data were used for training. In addition, the mean RMSE of the leave-one-subject-out cross-validation (CV) is shown. Joint moments and the anterior-posterior (A-P) and vertical ground reaction force (GRF) are normalized to bodyweight (BW) and bodyheight (BH).

Figure 6 summarizes the overall results for the cases where the number of training subjects was decreased from seven to four and to two subjects. Reducing the amount of training samples led to higher RMSE values except for the hip angle when training with four instead a seven subjects. Simulated data improved the results for joint angles independent of the amount of training subjects. When increasing the dataset by 16 times, the RMSE of hip, knee, and ankle angle decreased by 17, 27, and 23% for training with all seven subjects. In the case of training with four subjects, the RMSE of the knee joint angle could even be reduced by 31%. Moreover, the RMSE of the hip and ankle joint angle was lower when training with simulated and measured data of four subjects compared to training with only measured data of seven subjects. The RMSE of the knee joint angle was lower when training with simulated and measured data of two subjects compared to training with only measured data of seven subjects. However, hip flexion moment was worse for all training data configurations using simulated data. The knee extension moment and vertical GRF improved using simulated data for testing all training data configurations. The RMSE of ankle moment and A-P GRF improved using simulated data, unless data of only two subjects was used for training. When doubling the dataset, the RMSE of knee and ankle moment and vertical GRF decreased by about 6% for training with all seven subjects. When increasing the dataset by four times, the RMSE of A-P GRF decreased by about 2% for training with all seven subjects. Adding more simulated data worsen the estimation of joint kinetics and GRFs.

We added heat-maps, like Figure 6, for the Pearson correlation coefficient to the Supplementary Material. When increasing the dataset by 16 times, Pearson correlation coefficients increased from 0.967 to 0.975 for the hip angle, from 0.988 to 0.992 for the knee angle, and from 0.956 to 0.976 for the ankle angle when training with all seven subjects. The correlations of kinetics were above 0.97 without using simulated data when training with all seven subjects, except for the hip moment with 0.94. Correlations above 0.90 can already be classified as excellent (Taylor, 1990) and are higher than previous work (Dorschky et al., 2019). Correlation coefficients only increased for knee joint moment from 0.970 to 0.971 and for vertical GRF from 0.983 to 0.985 when adding simulated data.

We added individual results of all subjects to the Supplementary Material comparing the RMSE, relative RMSE (Ren et al., 2008), and the Pearson correlation coefficient for a different amount of simulated data. We differentiated between walking and running to allow a better comparison to other work which only focuses on walking or running.

## 4. Discussion

In this work, we presented a machine learning approach to extract joint angles, joint moments, and GRFs from a combination of simulated and experimental inertial sensor data. The goal was to combine the benefits of physics-based and data-driven approaches: We used simulated data from a physics-based model to reduce exhaustive collection of training data and used this to train data-driven models which can provide low-latency feedback on biomechanics.

The simulated data decreased the generalization error (here RMSE) of the joint angles by up to 31%. Pearson correlation coefficients of joint angles were already between 0.96-0.99 without using simulated data and were ≥0.98 with simulated data. Simulated data had a greater effect on RMSE than on correlation coefficients as the RMSE is more sensitive to outliers, and simulated data improved especially the results of outlying subjects. For example, the RMSE of the knee angle improved by 38% for S03 whose ankle dorsiflexion angle was smaller at toe-off compared to the other subjects (compare Figure 4 and Figure 5). For joint moments, the simulated data decreased the generalization error only partly when estimations based on measured data were above average (i.e., above the mean CV error). Simulated data worsened the performance for hip joint moment estimates. This could be explained by the discrepancy between simulated hip joint moments and its reference. This difference is visible in Figure 4, which shows noisy oscillating joint moments for the simulations. One reason may be that only joint angles and GRFs, and no joint moments, were tracked by the musculoskeletal model in Equation 1. Thus, the model tried to follow the predefined joint angles and GRFs using unrealistic (min-max switching) muscle activation patterns. This likely led to the noisy joint moment estimations. A higher weighting of the effort term in the optimal control simulation might lead to smoother muscle activations and thus muscle forces and joint moments. Joint moments could also be tracked in the optimal control simulations. However, the results for joint angles and GRFs might get worse. Another reason may be that the reference joint moments are too smooth, as filtering of marker data and force plate data was applied before computing joint moments (Dorschky et al., 2019). Overall, the reference joint moments were not directly measured but estimated using inverse dynamics. Thus, error accumulation lead to inaccuracies especially for the hip joint moment.

The estimation result of GRFs was already better without simulated data compared to previous work with Pearson correlations >0.97. In Dorschky et al. (2019), the RMSE of A-P and vertical GRF was 5% BW and 15% BW. In this work, the mean RMSE of A-P and vertical GRF was about 3% BW and 10% BW using only measured data (analyzing the GRFs over the complete cycle). The simulations were created using the same musculoskeletal model as in Dorschky et al. (2019), who reported errors in the estimation of GRFs and ankle joint moments due to model inaccuracies, as the foot was modeled with a single rigid segment. Consequently, simulated data only partly enhanced the estimation of GRFs and ankle joint moments in Tables 3, 4.

A direct comparison to previous work is difficult as different datasets of varying number of sensors, sensor positions, subjects, and movements were used for evaluation. Machine learning models dedicated to one single task, for example, for estimating single joint angles or specialized for walking only, will probably outperform our machine learning models which were jointly tuned for different output variables. In order to fairly compare different approaches, they would all need to be tested using the same datasets. The presented machine learning approach outperforms our previous physics-based approach (Dorschky et al., 2019) evaluated on the same data-set. In contrast to physics-based approaches, machine learning models require representative training data. Combining simulated and measured data seems a promising approach (Mundt et al., 2020a). In this work, we focused on the comparison between learning on measured and learning on simulated data to evaluate whether simulations can decrease the generalization error by incorporating variations of movement. Future work should expand this method to 3D analysis and evaluate against state of the art methods (Stetter et al., 2019; Mundt et al., 2020a). 3D biomechanical optimal control simulations are more expensive to compute, but are advancing recently (Falisse et al., 2019).

The network architecture was specialized for pre-segmented walking and running cycles and a fixed input and output dimension. The segmentation and sampling was chosen to match with the simulation with a fixed number of collocation nodes. We trained the CNNs separately in order reduce the output dimension and consequently the amount of trainable parameters in the network to avoid overfitting. It has been shown that individual CNNs can outperform bigger networks with multiple output variables (Hannink et al., 2017). However, the first layers of the different networks, which act as feature extractors, probably share some common features such that multi-task learning or transfer learning might improve results (Caruana, 1997). Future work should consider different network architectures which avoid pre-processing (segmentation into walking and running cycles and resampling) of sensor data like fully (circular) convolutional networks and allow a continuous estimation of movement biomechanics using recurrent architectures like long short-term memory networks (Mundt et al., 2020b). In addition, the feature extraction using convolutional layers should be explored. In the CV, two dimensional convolution yielded superior results compared to one dimensional convolutions over time which are typically used for inertial sensor data (Hannink et al., 2017). The 2D convolution was applied over time and over adjacent sensor axes, where data is likely to be correlated. The order of sensor axes was not optimized and data of accelerometers and gyroscopes were not split, although different feature extractors for different sensor types may yield better results. As CNNs were tuned on measured data, we assume that different architectures would not influence the comparison between learning on measured and simulated data.

A reality gap was apparent between simulated and measured inertial sensor data. Simulated inertial sensor data were less noisy than measured data (e.g., Figure 3 longitudinal acceleration of foot sensor). We modeled a rigid attachment of virtual sensors on the musculoskeletal model. In reality, the connection is loose due to soft tissue, which could be considered by a wobbling mass model. Another option is to use domain adaptation learning. For example, generative adversarial networks could be trained to learn a mapping between simulated and measured data (Shrivastava et al., 2017). In preliminary work, we learned a direct mapping between simulated and measured data using supervised learning. This yielded worse results which might be explained because end-to-end learning is typically superior. Further investigations and evaluations are necessary here.

In this work, we jointly learned from simulated and measured data. In our case, this approach worked better than training on simulated data and fine-tuning on real data. We assume that robust features were learned which were invariant to noise and movement artifacts. However, overfitting to simulated data was observed, for example for the vertical GRF where the performance decreased when adding three to fifteen times as much simulated as measured data. Instead of random sampling (see section 2.2), simulated data could only be created for those points where the current model is least certain. Thus, outliers could be covered with simulated data, whereas the performance of data that lies within the measurements would ideally not be affected. Future work should consider methods, where simulated data is generated iteratively during training within a closed loop. For example, Ruiz et al. (2018) proposed a meta-learning algorithm to learn how to simulate. The algorithm should adjust parameters of a simulator to generate synthetic data such that a machine learning model achieves a higher accuracy.

Data augmentation is commonly used to artificially expand a data set for training deep neural networks, but most approaches use only label-preserving transformations of input data (e.g., adding noise or rotating sensor axes, Um et al., 2017). In contrast, the presented method creates new pairs of input and output data such that a wider range of movement mechanics is covered. In this work, we generated the simulated data based on the training data distribution of the individual training subjects to take into account intra-subject variability. The simulated data filled the sparsely populated space of measured training data, as more variations of movements and speeds were included in the training set. This can be seen in Figure 4 where the simulated data covers a wider range of biomechanics and less space between curves is apparent. However, on the one hand not all test data is covered within the simulated and measured data (see e.g., maximum knee extension moment) because of inter-subject variability. On the other hand, we surmise that the simulated data was spread too widely for GRFs as the estimated variance was high especially for initial contact. When we used simulated data closer to the mean of measured data for training the CNNs, the estimation of joint moments and GRFs was slightly better, but the estimation of joint angles was slightly worse. Future work may consider to use more light-tailed data distributions than multivariate normal distributions.

Results depended on the training data distribution. For example, the hip angle improved when training with four instead of seven subjects, likely because the testing data distribution better matched that of the training data distribution of the four subjects. To cover a wider range of movement variations and to achieve a representative dataset, different data sources could be combined using the biomechanical simulation. Public datasets of movement biomechanics could be tracked with the musculoskeletal model to obtain corresponding inertial sensor data. Instead of tracking joint angles and GRFs, video data or inertial sensor data could be tracked with the model (Heinrich et al., 2014; Dorschky et al., 2019). This shows the potential of using optimal control simulations to create labeled training data (corresponding inertial sensor data and biomechanics). Simulated inertial sensor data at different sensor positions could easily be obtained.

While the recording of measured data (without post-processing) took about two weeks, it only took a few hours to create the same amount of ready-to-use simulated data with the implemented simulation framework. As shown in Figure 6, the estimation of joint angles was even better using a reduced dataset with simulated data compared to using all measured data without simulated data. On the one hand, using simulated data increases the number of samples and thus minimizes the risk of overfitting. On the other hand, simulated data includes additional variations of movement such that unseen data is covered with a higher probability. Simulated data would be of great advantage for rare events and abnormal movements where training data is hard to acquire, for example, for detecting an impending fall. Overall, biomechanical simulations can supplement time-consuming and expensive data collections to achieve a better generalization of machine learning models.

In summary, we presented a novel approach to generate an (in principle) infinite set of inertial sensor data with corresponding biomechanical variables using optimal control simulations of walking and running. We evaluated training on simulated data compared to solely learning on measured data. The simulated data improved the estimation of joint angles. The simulation-aided estimation of joint moments and GRFs was limited by inaccuracies of the musculoskeletal model. Improving the physics-based model or domain adaptation learning may help to reduce the gap between real and simulated data. The current method is a first step of using optimal control simulation for training deep neural networks and was evaluated for sagittal plane biomechanics only. In future work, this method should be evaluated for 3D biomechanical analysis. In addition, different datasets could be combined using the optimal control simulation in order to create representative datasets of human movement.

In conclusion, machine learning can benefit from available domain knowledge on biomechanical simulations to supplement cumbersome data collections. This enables the training of robust data-driven models that can provide real-time feedback on biomechanics “in the wild,” for example, to reduce injury risk, for rehabilitation movement training, or for controlling active assisting devices such as exoskeletons.

## Data Availability Statement

Please contact the corresponding author to request the datasets.

## Author Contributions

ED performed the biomechanical simulations and trainings of the neural networks and wrote the paper. MN and AB supported the implementation of the biomechanical simulations. CM supported the conception end evaluation of the machine learning approach. AK and BE supervised the overall conception and design of the work. All authors reviewed the paper and approved the final manuscript.

## Conflict of Interest

The authors declare that the research was conducted in the absence of any commercial or financial relationships that could be construed as a potential conflict of interest.
