# Supplementary material for: CNN-Based Estimation of Sagittal Plane Walking and Running Biomechanics From Measured and Simulated Inertial Sensor Data
Source: Front Bioeng Biotechnol. 2020 Jun 26;8:604. doi: 10.3389/fbioe.2020.00604 (PMC7333079; doi:10.3389/fbioe.2020.00604)
Supplement: Supplementary file 1 [file Data_Sheet_1.PDF]

Supplementary Material

1 IMPLEMENTATION

The following Python code was used to define the Convolutional Neural Networks using Keras (Chollet, 2015).

```
def get_model(param):
    """Generate a model from a parameter set

    Parameters
    ----------
    param : list
        Parameters of the network: list of dicts, where 1 dict describes 1 network layer.

    Returns
    -------
    keras.engine.sequential.Sequential
        A sequential Keras model.
    """

    # extract params for layer 1
    filters1 = param[0]['filters']
    (kernel_size11,kernel_size12) = param[0]['kernel_size']
    strides1 = param[0]['stride']
    (pool_size11, pool_size12) = param[0]['pool_size']
    # extract params for layer 2
    filters2  = param[1]['filters']
    (kernel_size21,kernel_size22) = param[1]['kernel_size']
    strides2 = param[1]['stride']
    (pool_size21, pool_size22) = param[1]['pool_size']

    model = Sequential()
    model.add(Conv2D(filters1,(kernel_size11,kernel_size12),strides=strides1, activation='relu',padding='same'));
    model.add(MaxPooling2D(pool_size=(pool_size11, pool_size12)));
    model.add(Conv2D(filters2,(kernel_size_21,kernel_size_22),strides=strides2, activation='relu',padding='same'));
    model.add(MaxPooling2D(pool_size=(pool_size21, pool_size22)));
    model.add(Flatten());
    model.add(Dense(nSamplesOut, activation='relu'));
    model.add(Dense(nSamplesOut, activation='linear',kernel_regularizer=regularizers.l2(0.001)));
    adam = optimizers.Adam(learning_rate=0.001, beta_1=0.9, beta_2=0.999, amsgrad=False);
    model.compile(loss='mse',optimizer=adam);

    return model
```

2 SUPPLEMENTARY RESULTS

This section provides additional results: Fig S1 shows the Pearson correlation coefficients for the cases where the number of training subjects was decreased from seven to four and to two subjects. Fig. S2 to Fig. S7 provide individual results of all subjects comparing the root-mean-square error (RMSE), relative RMSE (Ren et al., 2008), and the Pearson correlation coefficient for a different amount of simulated data. We differentiated between walking and running to allow a better comparison to other work which only focuses on walking or running.

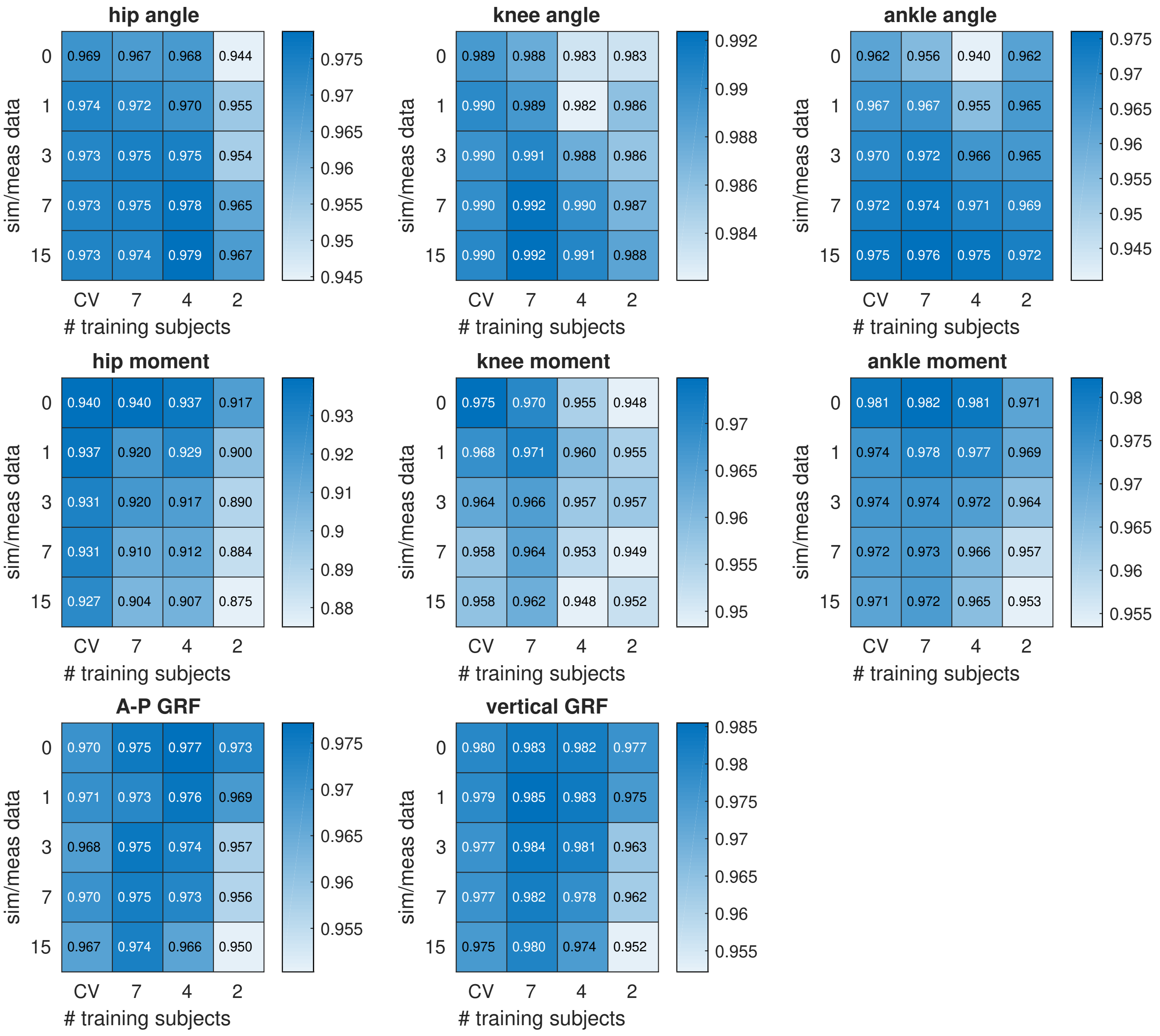

Figure S1. Overall results of the Pearson correlation coefficient for the estimated sagittal plane biomechanical variables. The vertical axis indicates the ratio between simulated (sim) and measured (meas) data used for training. The horizontal axis indicates the number of training subjects whose data were used for training. In addition, the mean Pearson correlation coefficient of the leave-one-subject-out cross-validation (CV) is shown. Joint moments and the anterior-posterior (A-P) and vertical ground reaction force (GRF) are normalized to bodyweight (BW) and bodyheight (BH).

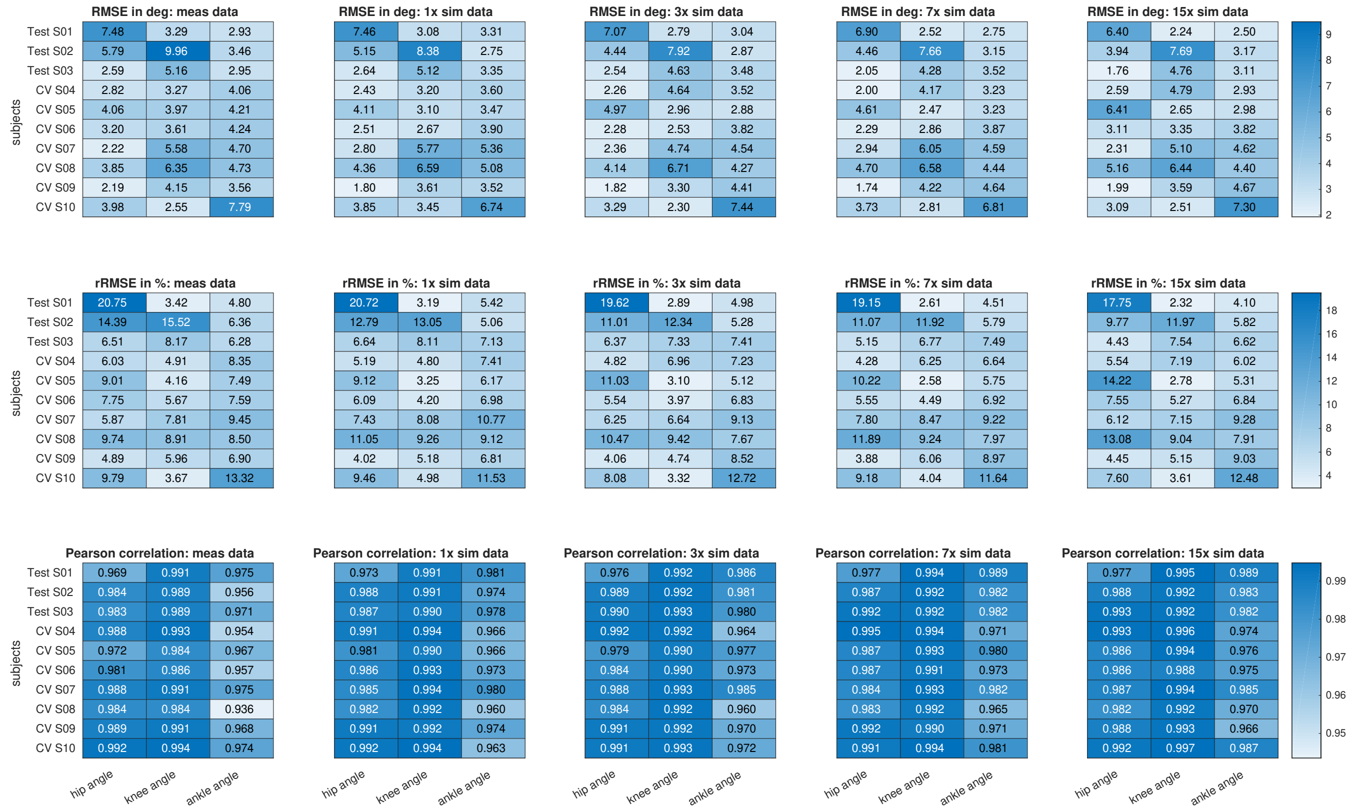

Figure S2. Results of walking joint kinematics for all, testing and cross-validation (CV), subjects: root-mean-square error (RMSE) of joint angles in deg, relative RMSE (rRMSE) in %, and Pearson correlation coefficient.

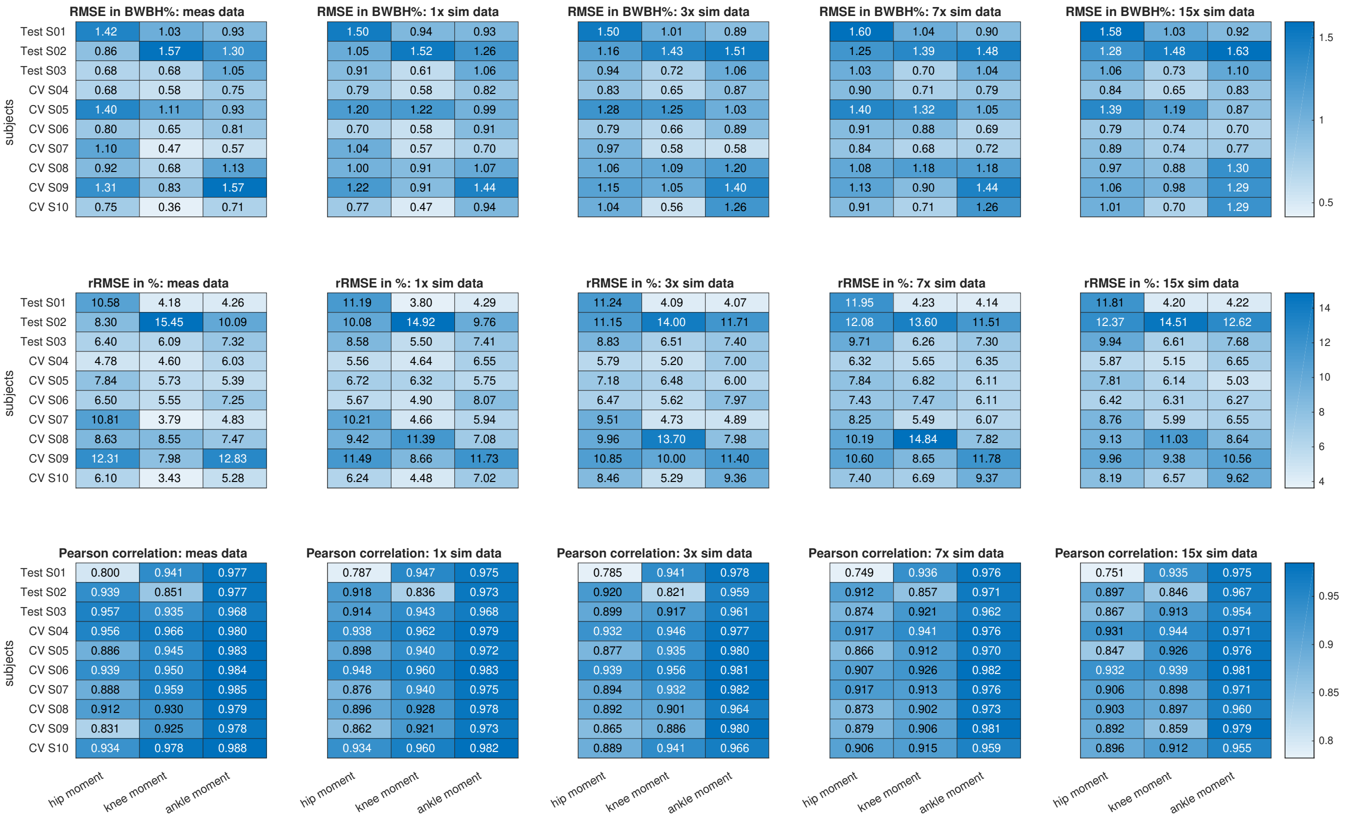

Figure S3. Results of walking joint kinetics for all, testing and cross-validation (CV), subjects: root-mean-square error (RMSE) of joint moments in BWBH%, relative RMSE (rRMSE) in %, and Pearson correlation coefficient.

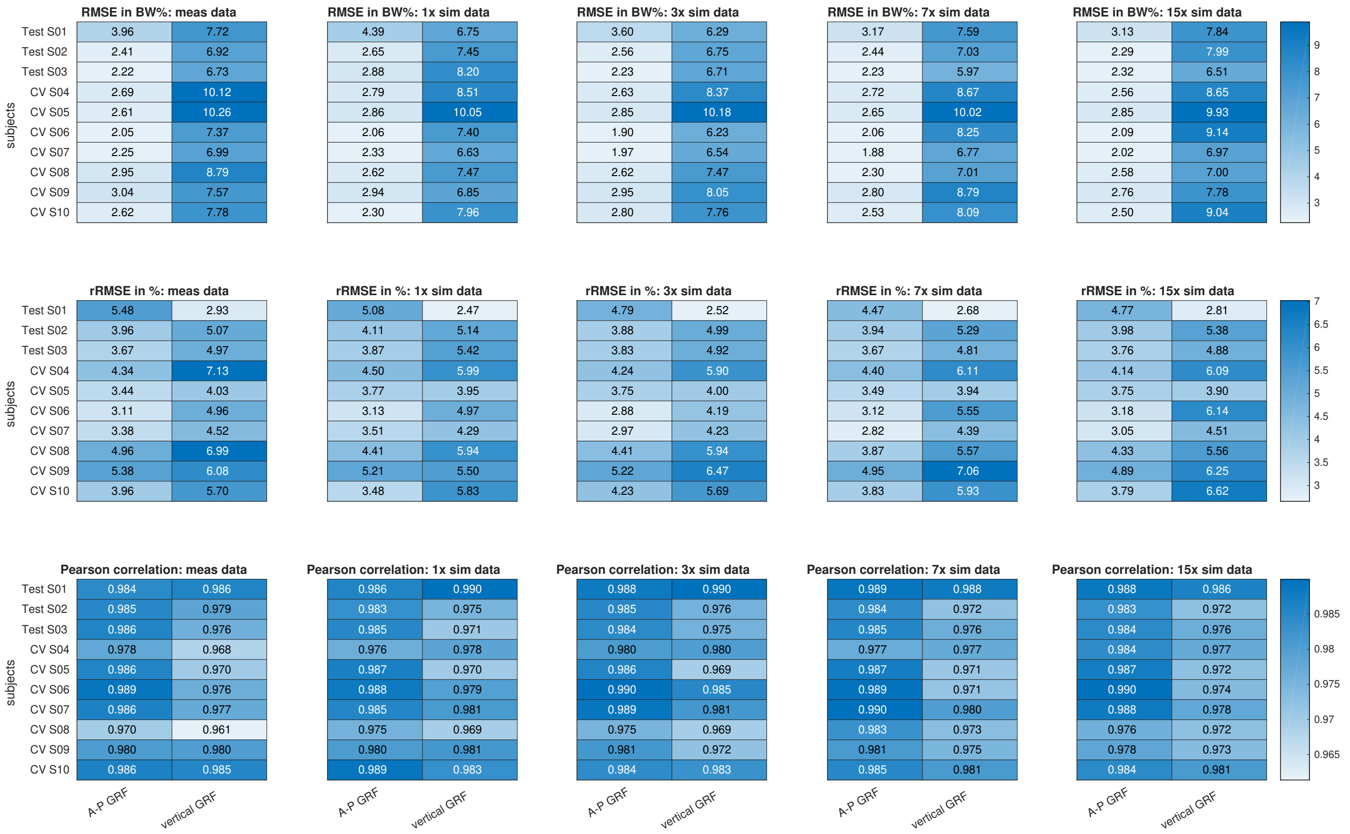

Figure S4. Results of walking ground reaction forces (GRFs) for all, testing and cross-validation (CV), subjects: root-mean-square error (RMSE) of GRFs in BW%, relative RMSE (rRMSE) in %, and Pearson correlation coefficient.

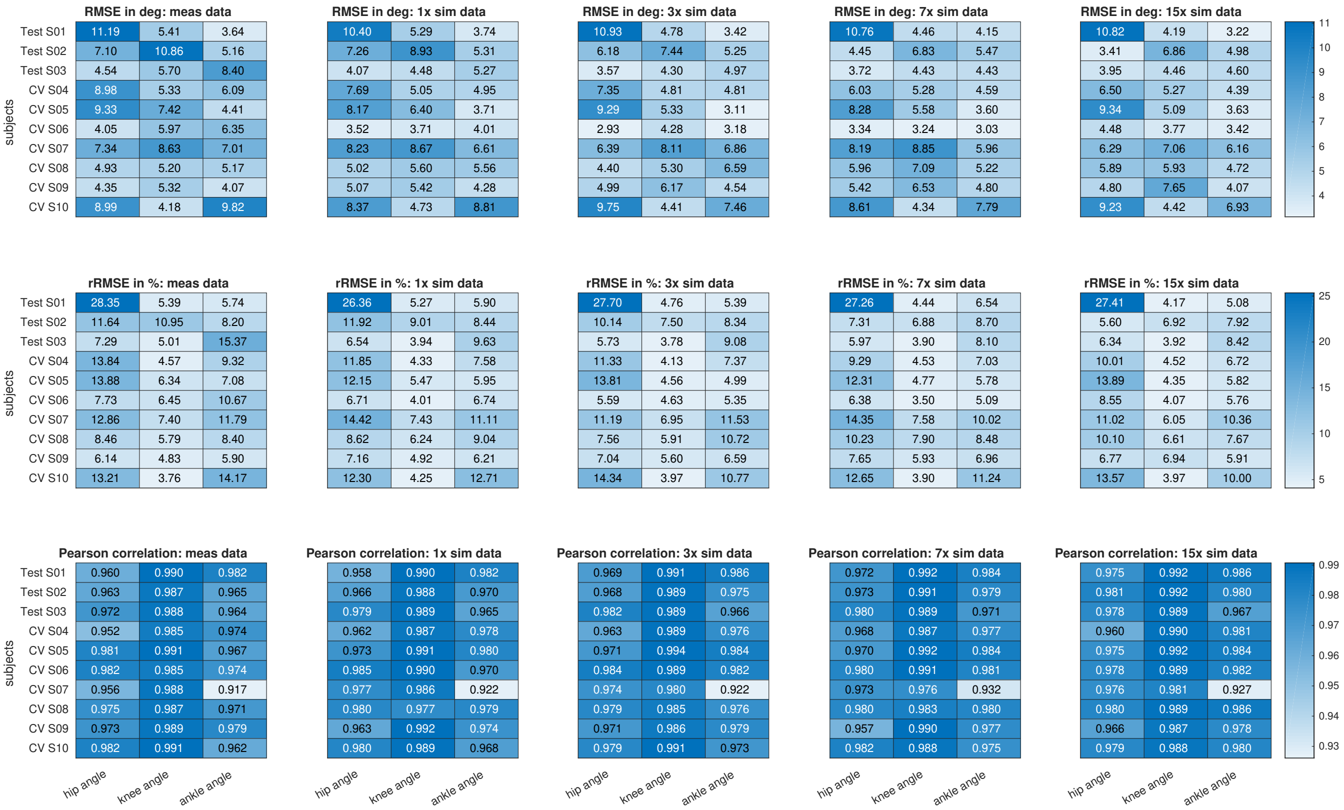

Figure S5. Results of running joint kinematics for all, testing and cross-validation (CV), subjects: root-mean-square error (RMSE) of joint angles in deg, relative RMSE (rRMSE) in %, and Pearson correlation coefficient.

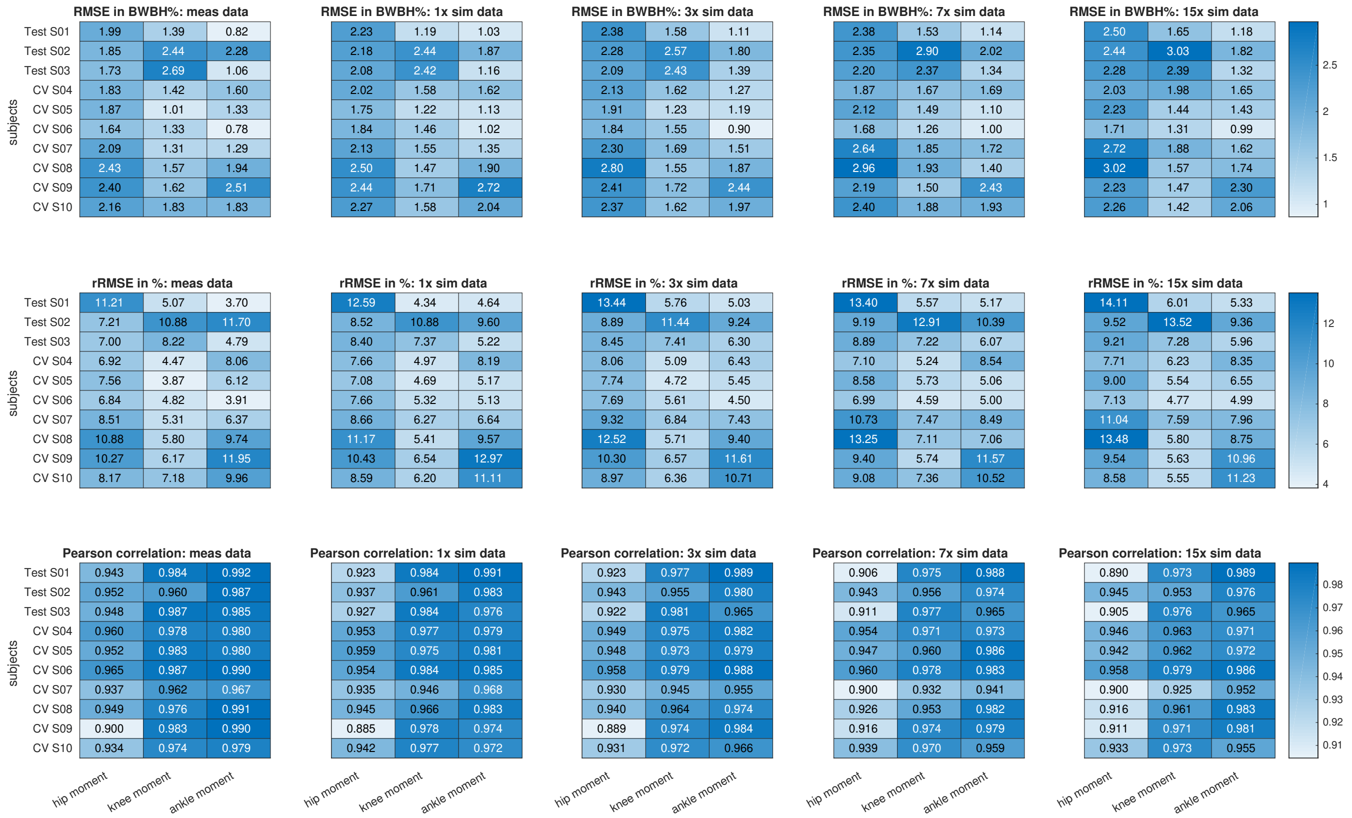

Figure S6. Results of running joint kinetics for all all, testing and cross-validation (CV), subjects: root-mean-square error (RMSE) of joint moments in BWBH%, relative RMSE (rRMSE) in %, and Pearson correlation coefficient.

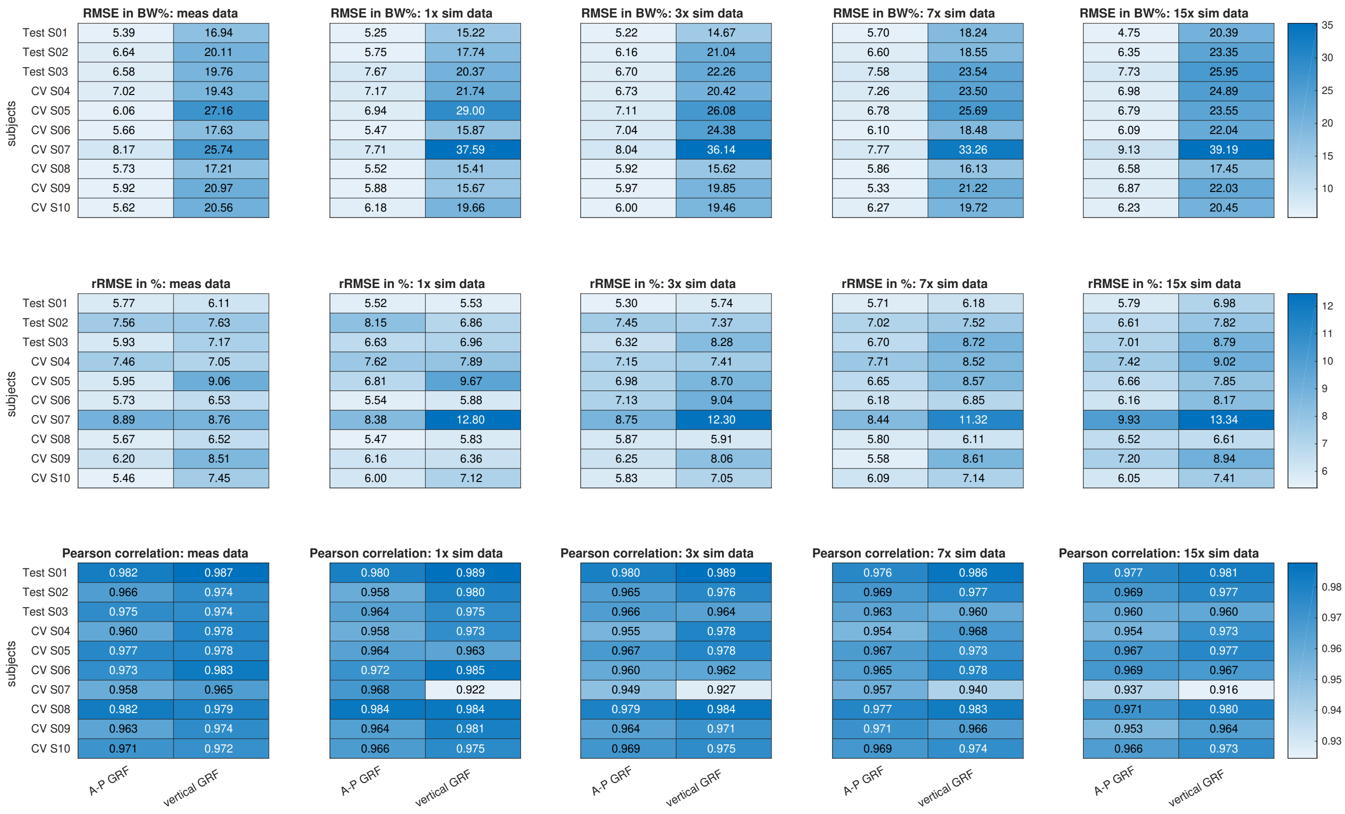

Figure S7. Results of running ground reaction forces (GRFs) for all, testing and cross-validation (CV), subjects: root-mean-square error (RMSE) of GRFs in BW%, relative RMSE (rRMSE) in %.

REFERENCES

[Dataset] Chollet, F. (2015). Keras. `https://github.com/fchollet/keras`. [Accessed April 22, 2020]

Ren, L., Jones, R. K., and Howard, D. (2008). Whole body inverse dynamics over a complete gait cycle based only on measured kinematics. *Journal of biomechanics* 41, 2750–2759
